# Digital Pain Assessment: Patient and Family Perspectives

**DOI:** 10.3390/nursrep16030092

**Published:** 2026-03-06

**Authors:** Rosemary Saunders, Kate Crookes, Kaoru Nosaka, Olivia Gallagher, Jeff Hughes, Caroline Bulsara, Max K. Bulsara, Seng Giap Marcus Ang, Beverley Ewens, Sue Haydon, Karla Seaman, Renée Graham, Debra Scaini, Karen Gullick, Michelle Gay, Christopher Etherton-Beer

**Affiliations:** 1 Centre for Research in Aged Care, School of Nursing and Midwifery, Edith Cowan University, Joondalup, WA 6027, Australia; 2 School of Psychological Science, The University of Western Australia, Crawley, WA 6009, Australia; 3 School of Health & Clinical Sciences, The University of Western Australia, Crawley, WA 6009, Australia; 4 PainChek^®^ Universal, 35 Lime St, Sydney, NSW 2000, Australia; 5 Curtin Medical School, Curtin University, Bently, WA 6102, Australia; 6 School of Nursing and Midwifery, The University of Notre Dame, Fremantle, WA 6160, Australia; 7 Institute for Health Research, The University of Notre Dame, Fremantle, WA 6160, Australia; 8 Hollywood Private Hospital, Nedlands, WA 6009, Australia; 9 Medical School, The University of Western Australia, Perth, WA 6009, Australia

**Keywords:** pain, aged, nursing, inpatients, surveys and questionnaires, pain measurement, family

## Abstract

**Background/Objectives:** Pain is a common symptom for hospitalised older adults. Pain is not always adequately assessed, which can lead to inadequate pain management and adverse patient outcomes. Thus, new technology-driven pain assessment tools have been developed; however, little is known about patients’ and families’ experiences of nurses using them in acute care. This study aimed to explore the perspectives of older adult inpatients and their families’ regarding nurses’ use of the digital technology-driven pain assessment application PainChek^®^ Universal. **Methods:** A survey was undertaken as part of a stakeholder evaluation of a randomised control trial exploring the effectiveness of nurse-led volunteer support and technology-driven pain assessment in improving the outcomes of hospitalised older adults. The PainChek^®^ Universal application was implemented on two medical wards of an acute private hospital in Western Australia as part of a larger single-centre, prospective, non-blinded, cluster-randomised control trial. This stakeholder evaluation invited older adult inpatients and their family members to participate in a survey about nurses’ use of the PainChek^®^ Universal application for pain assessment. **Results:** A total of 96 inpatients and 27 family members completed the survey. Thirteen patients and nine family members provided additional feedback. Over 90% of patients and family members agreed that the use of the PainChek^®^ Universal application was a positive addition to pain assessments, rendered no concerns, and helped nurses complete pain assessments. A total of 84% of patients and 87% of family members felt PainChek^®^ Universal provided a more accurate pain assessment. Survey feedback related to PainChek^®^ Universal application use, integration of technology, and need for further education. **Conclusions:** The findings suggest that older adults and their families recognised the benefits of nurses using a digital application for pain assessments. Technology integration in healthcare must be accompanied by patient and family education.

## 1. Introduction

Pain is a prevalent symptom for hospitalised patients globally [1,2,3]. Within the older adult population (65 years and older), pain rates have shown a comparative increase over time [4] and are a common and increasing concern [5,6]. For older adult patients, appropriate pain assessments are critical in guiding and evaluating the impacts of pain management and interventions [5]. However, research has consistently shown that assessment of pain, the use of validated pain assessment tools, and documentation of pain by nurses are inconsistent and inadequate, which can impact pain management, particularly for older adults [7,8,9,10,11,12]. Suboptimal pain assessment and management can lead to adverse physical and psychological patient outcomes, including reduced daily functioning and mobility, falls, depression and anxiety, stress, fear, feelings of powerlessness, sleep impairment, delirium, and behavioural symptoms, as well as prolonged hospital stays [11,13,14]. Suboptimal pain assessment and management can impact patient–nurse relationships [11]. A lack of education and explanation by healthcare professionals about patient care needs can hinder the ability to make empowered and collaborative care decisions that involve both the patient and family [15]. Patients have reported that they feel pain assessments completed by nurses have been inadequate and lacking in patient-centred care (PCC) [11,16]. Harmon et al. [11] found that nurses did not adequately explain pain-based numerical rating scales to older adult participants, which made it difficult for patients to accurately report their pain scores, leading to a void in PCC. Older people can find it difficult to quantify their pain verbally [11]; thus, the use of more innovative, observational pain assessment tools is pertinent.

The digital health, evidence-based technology solutions, and artificial intelligence (AI) have been identified as important innovations to help improve pain assessment and pain management, especially for those who are older adults and have cognitive impairment or limited verbal skills [17,18,19]. PainChek^®^ Universal has pioneered the use of digital-technology-based pain assessment applications as innovative interventions to enhance pain assessment practices [20]. PainChek^®^ Universal is the world’s first clinically validated medical device for the assessment of pain [20]. PainChek^®^ Universal includes technology-based pain assessment as part of patient care, alongside a digital platform to analyse, record and facilitate healthcare team collaboration [20]. The PainChek^®^ Universal app has been implemented across 1700 older adult care facilities globally, where it has been positively evaluated as enabling best-practice pain management [21]. As PainChek^®^ Universal is being introduced in acute care settings, understanding older adults’ and family members’ levels of app acceptability and experience is valuable.

It is important that older adults as participants, consumer representatives, and research team members are invited to participate in research [22]. There is limited research on the inclusion of older adults in the development of digital tools for pain assessment; however, there have been two recent studies on pain assessment that sought older adult perspectives [23,24]. Lopes et al. [23] included older adult participants’ feedback within a larger research cohort to validate the content and user experience of a chronic pain assessment app to refine the prototype. In the development of a self-managed pain management app, older adult participants were also consulted on the interface, usability, app functionality, educational resources, and suggestions for additional medically focused educational material to help evaluate the digital tool’s development [24]. Participants’ engagement in design processes resulted in user perceptions of the digital tools as being useful and easy to engage with [23,24]. Although older adults have participated in the curation of digital tools [23,24], little is known about the family experiences of a patient’s pain assessments [25]. There is no reported study of patients’ or families’ perceptions of nurses using a technology-driven pain assessment app. Therefore, this study reports the stakeholder evaluation (i.e., older adult inpatients and family members) of the technology-driven pain assessment component of a larger study entitled the “Effectiveness of nurse-led volunteer support and technology-driven pain assessment in improving the outcomes of hospitalised older adults: Cluster randomised control trial” [26].

## 2. Materials and Methods

### 2.1. Design

A stakeholder evaluation using a cross-sectional survey was utilised as part of single- centre, prospective, non-blinded, cluster-randomised controlled trial.

### 2.2. Setting

This study was conducted from February 2021 to July 2022 across four medical wards in the largest acute private hospital in Australia. The hospital had over 900 beds that provided a range of acute medical, surgical, and rehabilitation services. The site was chosen due to the large number of older adult patients admitted to the hospital, and one site was deemed adequate based on the research design.

### 2.3. Sample and Recruitment

The sample included participants who had been recruited to the two intervention wards using PainChek^®^ Universal (PainChek Ltd., Sydney, Australia) as part of the main study [26]. Cluster randomisation occurred at the level of the ward, and the sample size of the main study was determined by a power calculation as reported in the study protocol [26]. Prior to discharge, eligible participants (patients n = 176) were informed about the stakeholder evaluation and invited to participate in this study. Participants provided written consent and the contact details of a family member so they could be invited to participate in a telephone stakeholder survey. The nominated family member (n = 156) was contacted via phone 48 h after discharge of the patient participant to enquire if they would participate.

### 2.4. Intervention

**Figure 1:**
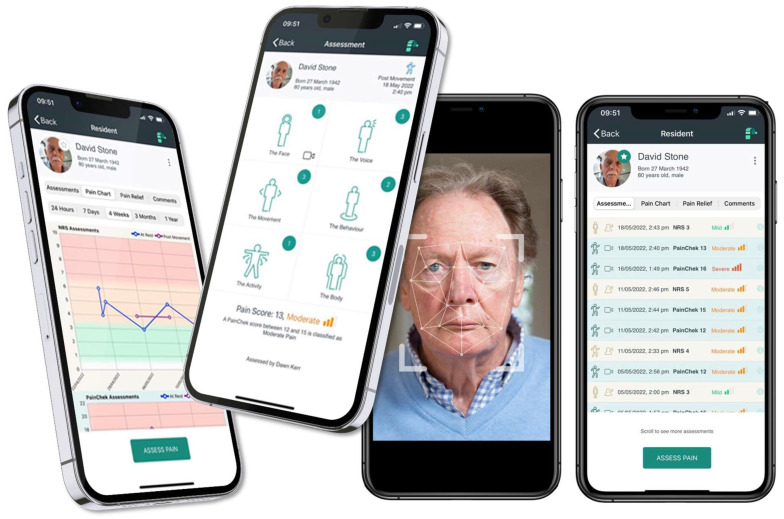
PainChek^®^ Universal digital pain assessment system.

The PainChek^®^ Universal app (Figure 1) has two components to support users in assessing pain. The first component is the Numeric Rating Scale (NRS), and the second component is the PainChek^®^ scale for people who are not able to reliably report their pain [27]. The PainChek^®^ scale utilises both automated facial detection and analysis, in combination with user inputs of behavioural changes, to produce a pain score [27]. As all participants were able to reliably report pain, the NRS component of PainChek^®^ Universal was used in this study. Patients were informed about the purpose of the PainChek^®^ Universal app that was used via an iPad and were encouraged to ask questions about it. Nursing staff undertook pain assessments and recorded the patient-reported pain score in the app. The functionality of PainChek^®^ Universal enables nurses and other health professionals to review the patients’ pain scores via the app dashboard and to analyse pain trends and responses to pain management interventions [27].

### 2.5. Data Collection

Inpatient participants completed a paper-based survey prior to discharge about their perceptions of nurses’ use of PainChek^®^ Universal.

Family members completed a telephone survey administered by research assistants and research team members (RG, RS, KC). Both the patient’s and family member’s surveys included an initial question about their awareness of the digital application in the ward. If they were not aware, they did not proceed with the survey. The survey included two demographic questions (age and gender), three statements about their perceptions (usefulness for assessment, accuracy of assessment, clinical utility for nurses to undertake pain assessment) of the PainChek^®^ Universal app, and one statement related to any concerns. The four items were developed based on a review of the literature and reviewed by a consumer.

An additional demographic question was included in the family members’ survey, which asked about their relationship to the patient.

All survey participants were asked to indicate their level of agreement to four statements about PainChek^®^ Universal using a 5-point Likert Scale. For patient participants, this scale ranged from 1 to 5, with “1” = “strongly disagree”, “2” = “disagree”, “3” = “neutral”, “4” = “agree”, and “5” = “strongly agree”. Family member participants were provided the same scale, with the addition of an “I don’t know” response option for all survey prompts. Both surveys included an open-ended question “Do you have any other feedback?”

### 2.6. Data Analysis

All statistical tests and analyses were conducted using SPSS Statistics 28 for Windows, and survey responses were analysed as ordinal data. This research treated all survey non-responses from patient participants and “I don’t know” responses from family participants as invalid responses and were excluded them from the analysis. Quantitative responses were analysed using descriptive and frequency statistics. Open-ended comments were analysed using content analysis [28].

### 2.7. Ethics

All inpatient participants were provided with a participant information form (PIF) and provided written consent. A PIF for family participants was provided to patients to give to their family members. At the time of the telephone interview, the researcher obtained verbal consent from the family member prior to commencing the telephone survey. This study was conducted in accordance with the Declaration of Helsinki and was approved by the hospital Human Research Ethics Committee (HREC) (number: 2057) and the university HREC (number: 2021-02210-SAUNDERS).

## 3. Results

### 3.1. Characteristics

**Figure 2:**
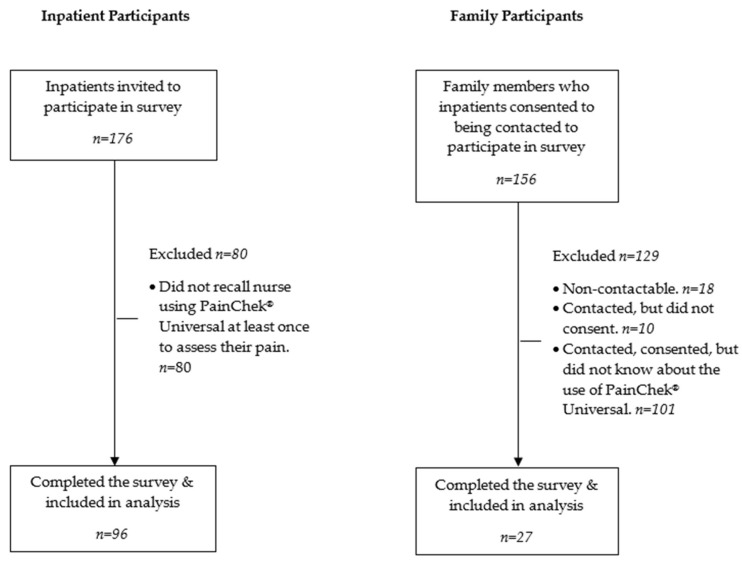
Participant recruitment, eligibility and follow-up.

A sample of 176 inpatients were invited to participate in the survey, and 156 family members of these patients were invited based on the consent to contact them provided by patient participants. Figure 2 shows the participant recruitment, eligibility and follow-up.

A total of 96 inpatients (response rate 54.5%) were included in this study as they stated they recalled the nurse using PainChek^®^ Universal to assess their pain at least once. Of the 156 family members whose details were provided by patient participants and eligible to participate based on being a family member of a patient in the study, 18 were non-contactable, 10 were contacted but did not consent to participate, and 101 consented but did not complete the survey because they were unaware of PainChek^®^ Universal being used. Thus, 27 family members (response rate 19.6%) who knew about the use of PainChek^®^ Universal responded to four survey statements related to their perceptions of PainChek^®^ Universal. Table 1 shows the demographic characteristics of the inpatient and family member survey respondents.

### 3.2. Perceptions of the Technology-Driven Pain Assessment Application

Inpatient and family survey results are summarised in Table 2 and Table 3. Most of the patient and family respondents “agreed” or “strongly agreed” that they had no concerns with PainChek^®^ Universal for pain assessment (92%). In addition, over 90% of patients and 100% of family members also “agreed” or “strongly agreed” that PainChek^®^ Universal had been a positive addition to the patient’s pain assessment. Furthermore, 91% of patients and 94% of family respondents “agreed” or “strongly agreed” that the technology-driven tool made it easier for nurses to complete pain assessments. The responses on the ability of PainChek^®^ Universal to provide a more accurate pain assessment achieved high proportions of “agree” and “strongly agree” with 84% of patients and over 87% of family members in agreeance, which is proportionally the lowest across the four survey statements for both cohorts. These results indicate a high level of patient and family acceptance of PainChek^®^ Universal being used to assess pain in older adult inpatients within an acute clinical setting.

### 3.3. Patient and Family Comments

Thirteen patients and nine family respondents provided comments to the open-ended question “Do you have any other feedback?” The feedback highlighted three main areas: PainChek^®^ Universal app use, technology use, and the need for patient and family education.

Both patients and family members expressed mostly favourable support of the PainChek^®^ Universal app being used in the wards. Patients shared that they felt PainChek^®^ Universal was a “good system” and “I think it’s a good idea.” One participant shared that “PainChek^®^ has given me confidence to go home.” A patient shared they were “…impressed by the person who did the assessment”. Patients perceived that PainChek^®^ Universal was “…very helpful for nurses” completing pain assessments, because “if it saves more time for the nurses, it is good.” One patient provided a useful suggestion “While collecting the overall pain score, it may be a good idea to collect specific pain region of the patient because the pain score for different body parts might be different.” The ability to collect regional pain data has been added as a new feature of the app [29]. A family member shared that they felt the use of PainChek^®^ Universal “…made Mum feel part of the process and care.” A further family participant shared that they trusted the use of PainChek^®^ Universal based upon the positivity of the nurses using the app; “if the nurses think it is a good idea to do then I support it!”

There were mixed sentiments shared about nurses using a digital app for pain assessment. Patients felt positive about the use of the PainChek^®^ Universal app because it is “good to promote technology” and it was perceived as normalised, “…it’s to go with the times.” In contrast, one patient stated, “people are the important part rather than the iPads.” Family respondents were concerned about the recording of private information in a digital app: “I am concerned that my Dad’s personal information will be shared over various technologies”, and another stated, “I am concerned about privacy of information when using the technology.”

The need for patient and family education on the use of digital devices and how pain assessments were completed using the PainChek^®^ Universal app was shared by both groups. One patient felt that “the PainChek^®^ app should be explained so that patient and volunteer understand.” The ambiguity of the pain assessment process and the use of technology were also described by family members. One family member stated that they “saw the staff use it [PainChek^®^ Universal], but didn’t really know much about it,” with another saying, “I don’t know why the iPads were used.” A further family respondent felt there was also a need for staff education in using the app, “the staff needs to be familiar to this function and that is part of the learning process.”

## 4. Discussion

This stakeholder evaluation focused on exploring and understanding older adult inpatients’ and their family members’ experiences and perspectives of nurses using the PainChek^®^ Universal app as part of pain assessments within an acute care setting. Overall, both patients’ and family members’ survey responses showed that PainChek^®^ Universal was positively viewed as part of pain assessment practices and had no significant concerns about the app, but they did provide constructive feedback. The results from both respondent groups suggest that the utilisation of PainChek^®^ Universal can contribute to pain assessment practices. Patient perspectives from other studies have highlighted the need for improvements in pain assessment to inform pain management [16].

Patients’ and families’ open-ended feedback showed that the use of the technology was mostly viewed as favourable and accepted. Our findings showed that most participants supported PainChek^®^ Universal being used to complete pain assessments This finding aligns with other digital assessment tools being evaluated positively by health professionals and patients. For example, the majority of healthcare providers and patients had positive perceptions and experiences of the vital signs app VinCense mobile in a primary care setting [30]. Furthermore, studies in both hospital and home care settings have yielded similar results, where participants felt technology and the use of devices were socially acceptable and positively affected their care [31,32]. Research on the integration of digital devices in care provision found that participants did not have negative feelings towards nurses using iPads [32]. Nurses’ use of iPads has the potential to optimise documentation, enhance communication, and support collaborative learning between the nurse and patient [32].

The importance of PCC was emphasised by only one patient stating, “people are the important part rather than iPads.” This comment is a critical point and an important reminder that pain assessment should involve the patient, and, if any technology is used as part of care delivery, it needs to be clearly explained to the patient and family [33]. Other studies found that nurses’ use of bedside touchscreen monitors to access patient records, results, and charts can impact the nurse–patient relationship, as participants felt nurses were sometimes divided in their ability to interact, communicate, or engage with patients [31]. Although not found in our research, the use of technology can result in fragmented interactions, loss of eye contact, and a sense of silence whilst inputting data into digital platforms, which increases levels of patient uncertainty, frustration, and inability to consistently engage with nurses [31,34,35]. Nurses should explain digital assessment processes to patients to ensure they understand the purpose and process of the assessment, as well as ensure nurses are listening and responding to patient concerns [34,35].

Open-ended comments from both patients and family members highlighted the need for patient and family information and education on the use of PainChek^®^ Universal as part of patient pain assessment using a digital device. Patients shared that “the PainChek^®^ app should be explained so that patient and volunteer understand” better. Furthermore, the need for clarification on the integration of technology was shared by two family members: “saw the staff use it [PainChek^®^ Universal], but didn’t really know much about it’; “I don’t know why the iPads were used.” These statements demonstrate a gap in nurses providing patient education about the tools, assessments and documentation whilst utilising a digital tool. If nurses do not educate the patient or client about why they are using an iPad or inputting information into a computer, it becomes difficult for the patients or family members to fully understand what the nurse is doing or the benefits of an app such as PainChek^®^ Universal [32,34]. It is essential for organisations to provide healthcare staff training on digital platforms and the tool’s benefits for care delivery as this education enables better patient and family education [35]. Furthermore, family and clinician education is vital to ensure that the functionality and accuracy of a device is understood [31]. Staff training also helps increase exposure, user confidence, and the ability to troubleshoot issues with digital tools [36,37], which is important as a family member shared that they felt the nurses needed more familiarity with PainChek^®^ Universal’s functionality.

Family members also shared concerns about patient data privacy, which is consistent with findings from other studies involving patients, family members, and healthcare professionals [30,38,39]. Healthcare organisations and providers can help mitigate these concerns by focusing on building trust and educating all consumer and staff stakeholders [40]. Educated staff are better able to provide effective patient education, which supports increased positive perceptions of technology integration within care delivery, education, and documentation, whilst further contributing to improved pain management outcomes [31,34,35]. It is important to note that PainChek^®^ does not store or share images and that PainChek^®^ Universal is compliant with privacy and cybersecurity requirements across several jurisdictions including Australia, New Zealand, the United Kingdom, Europe and North America [41].

Including families and patients in the stakeholder evaluation provided valuable feedback. It especially highlighted the need for providing information to patients and families about the technological devices that are used as part of care, and there needs to be consideration of education specific to these groups. Additionally, training for nurses and other health professionals about the app needs to include content on patient and family education.

## 5. Limitations

This study was limited to participants from a single hospital and therefore generalisability is limited. However, the results of this study contribute to a growing body of older adult research focused on inpatients’ and families’ experiences with digital pain assessment apps. The number of eligible family participants was notably small, with only 27 of the 128 participating as 101 had no knowledge of PainChek^®^ Universal being utilised. The small number of family respondents may be related to COVID-19 restrictions at the time of this study, which limited hospital visitors and their ability to observe nurses using PainChek^®^ Universal. This evaluation was also limited to experiences of the NRS component of PainChek^®^ Universal for patients who could reliably self-report pain. It is vitally important to understand the acceptability and experiences of family members when the PainChek^®^ component is used to assess pain of patients who are unable to self-report pain, Additional research utilising digital pain assessment tools across multiple areas within a hospital setting, as well as community or outpatient services, is warranted.

## 6. Conclusions

Pain assessment using appropriate tools for hospitalised older adults is an essential part of care. The technology-driven pain assessment app, PainChek^®^ Universal, was positively understood and accepted by the older adult inpatient and family member study respondents. The integration of technology in healthcare must be accompanied by education for patients and families, as they expressed the need to better understand and engage with digital apps for pain assessment.

## Figures and Tables

**Table 1:** Inpatient and family member demographic characteristics.

Variable	Inpatients	Families
Total n	n = 96	n = 27
	n (%)	
Gender		
Female	60 (62.5)	20 (74.1)
Male	36 (37.5)	7 (25.9)
Age		
40–49 y	0	4 (14.8)
50–59 y	0	4 (14.8)
60–69 y	6 (6.3)	10 (37.0)
70–79 y	25 (26.0)	7 (25.9)
80–89 y	47 (49.0)	1 (3.7)
>90 y	13 (13.5)	1 (3.7)
No response	5 (5.2)	0 (0.0)
Relationship to a patient		
Child	-	13 (48.1)
Spouse	-	13 (48.1)
Other immediate family ^a^/relative	-	1 (3.7)

Note. ^a^ Other immediate family included grandchildren, siblings, and in-laws (daughter and son).

**Table 2:** Inpatients’ perceptions of digital-technology-based pain assessment application.

Statement No.	1 Strongly Disagree	2 Disagree	3 Neutral	4 Agree	5 Strongly Agree	IC ^a^	Median (IQR) ^b^
	n (%)						
1. I had no concerns with PainChek^®^ Universal for assessing my pain (n = 91)	1 (1.1)	0 (0.0)	6 (6.6)	11 (12.1)	73 (80.2)	5	5.00 (5.0–5.0)
2. I think PainChek^®^ Universal was a positive addition to my assessments (n = 91)	1 (1.1)	1 (1.1)	7 (7.7)	16 (17.6)	66 (72.5)	5	5.00 (4.0–5.0)
3. Using PainChek^®^ Universal made it easier for nurses to complete pain assessments (n = 91)	1 (1.1)	0 (0.0)	7 (7.7)	13 (14.3)	70 (76.9)	5	5.00 (5.0–5.0)
4. PainChek^®^ Universal can provide a more accurate assessment (n = 90)	3 (3.3)	0 (0.0)	11 (12.2)	11 (12.2)	65 (72.2)	6	5.00 (4.0–5.0)

Notes. ^a^ Invalid count includes non-responses from patients and “I don’t know” responses from families and were excluded from the analysis. ^b^ Interquartile range reporting—values at 25% and 75%.

**Table 3:** Family members’ perceptions of digital-technology-based pain assessment application.

Statement No.	1 Strongly Disagree	2 Disagree	3 Neutral	4 Agree	5 Strongly Agree	IC ^a^	Median (IQR) ^b^
	n (%)						
1. I had no concerns with PainChek^®^ Universal for assessing the patient’s pain (n = 26)	0 (0.0)	2 (7.7)	0 (0.0)	7 (26.9)	17 (65.4)	1	5.00 (4.0–5.0)
2. I think PainChek^®^ Universal was a positive addition to patient assessments (n = 25)	0 (0.0)	0 (0.0)	0 (0.0)	7 (28.0)	18 (72.0)	2	5.00 (4.0–5.0)
3. Using PainChek^®^ Universal made it easier for nurses to complete pain assessments (n = 18)	0 (0.0)	0 (0.0)	1 (5.6)	8 (44.4)	9 (50.0)	9	5.00 (4.0–5.0)
4. PainChek^®^ Universal can provide a more accurate assessment (n = 16)	0 (0.0)	0 (0.0)	2 (12.5)	10 (62.5)	4 (25.0)	11	4.00 (4.0–5.0)

Notes. ^a^ Invalid count includes non-responses from patients and “I don’t know” responses from families and were excluded from the analysis; ^b^ interquartile range reporting—values at 25% and 75%.

## Data Availability

The original contributions presented in this study are included as aggregated data in this article. Further inquiries can be directed to the corresponding author.

## Public Involvement Statement

A consumer representative, from the hospital consumer advisory committee, was involved in this study as a co-researcher and contributed to the development of this study, discussion of findings, and recommendations. The consumer also actively contributed to the development of a video as part of educational resources in using the application.

## Guidelines and Standards Statement

This manuscript was drafted against the Strengthening the reporting of observational studies in epidemiology (STROBE) checklist for cross-sectional research [42].
